# Hopes and challenges for giant panda conservation under climate change in the Qinling Mountains of China

**DOI:** 10.1002/ece3.2650

**Published:** 2016-12-20

**Authors:** Minghao Gong, Tianpei Guan, Meng Hou, Gang Liu, Tianyuan Zhou

**Affiliations:** ^1^ Research Institute of WetlandBeijing Key Laboratory of Wetland Services and RestorationChinese Academy of ForestryBeijingChina; ^2^ Mianyang Normal UniversityMianyangSichuanChina; ^3^ Academy of Forestry Inventory and PlanningState Forestry AdministrationBeijingChina

**Keywords:** climate change, giant panda, habitat fragmentation, habitat shift, Qinling Mountains

## Abstract

One way that climate change will impact animal distributions is by altering habitat suitability and habitat fragmentation. Understanding the impacts of climate change on currently threatened species is of immediate importance because complex conservation planning will be required. Here, we mapped changes to the distribution, suitability, and fragmentation of giant panda habitat under climate change and quantified the direction and elevation of habitat shift and fragmentation patterns. These data were used to develop a series of new conservation strategies for the giant panda. Qinling Mountains, Shaanxi, China. Data from the most recent giant panda census, habitat factors, anthropogenic disturbance, climate variables, and climate predictions for the year 2050 (averaged across four general circulation models) were used to project giant panda habitat in Maxent. Differences in habitat patches were compared between now and 2050. While climate change will cause a 9.1% increase in suitable habitat and 9% reduction in subsuitable habitat by 2050, no significant net variation in the proportion of suitable and subsuitable habitat was found. However, a distinct climate change‐induced habitat shift of 11 km eastward by 2050 is predicted firstly. Climate change will reduce the fragmentation of suitable habitat at high elevations and exacerbate the fragmentation of subsuitable habitat below 1,900 m above sea level. Reduced fragmentation at higher elevations and worsening fragmentation at lower elevations have the potential to cause overcrowding of giant pandas at higher altitudes, further exacerbating habitat shortage in the central Qinling Mountains. The habitat shift to the east due to climate change may provide new areas for giant pandas but poses severe challenges for future conservation.

## 1 Introduction

Climate change since the Last Glacial Maximum has affected wildlife distributions and changed the habitat structures and functions of many species (Maclean & Wilson, 2011; Roberts, Nielsen, & Stenhouse, 2014). To avoid extirpation due to ongoing climate change, some species adapt to changing habitats in situ, some shift to colonize new areas, and those with poor dispersal ability become locally extinct (Chen & Thomas, 2011; Thomas, 1996). Range‐restricted, high‐altitude species are particularly vulnerable to changing climatic conditions because they are commonly characterized by poor dispersal ability, limited food resources, and historically driven habitat fragmentation (Crabtree & Ellis, 2010). This is the case for giant pandas (*Ailuropoda melanoleuca*) because of their exclusive bamboo diet, narrow habitat range, low reproductive rate, and small population size (Li et al., 2015; Wang, Ye, Skidmore, & Toxopeus, 2010).

Climate change may result in habitat loss and fragmentation and synergistically determine the shifting direction of population ranges, which degrade species fitness at the habitat and/or genetic levels (Brook, 2008; Peacock, 2011; Pyke, 2008). Many studies have projected the dynamics of habitat suitability under climate scenarios to uncover the role of climate change in shaping the distribution of giant pandas and found bamboo shortages, habitat loss, and northward shifts in population range (Fan et al., 2014; Li et al., 2014; Songer, Delion, Biggs, & Huang, 2012; Tuanmu et al., 2013). Few studies have quantified habitat shifts and patterns of habitat fragmentation induced by climate change despite this being a critical component to understanding the impacts of global change on giant pandas. Most calculations of species extinction risk assume that endangered species go extinct when they no longer have suitable habitats because of climate change (Hole et al., 2009). Species that depend on one specific plant species for survival and reproduction are expected to be more vulnerable to habitat fragmentation, and the giant panda is a typical example (Piessens, Adriaens, Jacquemyn, & Honnay, 2009; Zhu, Hu, Qi et al., 2013). Therefore, due to the risk of habitat loss and fragmentation, there is an urgent need to estimate giant panda habitat spatial patterns and fragmentation to clarify key areas and strategies for future conservation planning. Additionally, data in previous studies associating giant panda presence with environmental and climatic variables were based on the results of the Third National Giant Panda Survey (TNGPS) completed in 2000, and need to be updated with the most recent survey data.

The projected rates of climatic change indicate that range shifts over the next century are likely to be great (Hole et al., 2009). The most recent IPCC‐CMIP5 climate scenarios and the Fourth National Giant Panda Survey (FNGPS) completed in 2012 provide a great opportunity to develop a complete understanding of the relationship between habitat spatial patterns and climate change using updated, high‐quality population and climate data. Because fringe ecological patches are more sensitive to environmental change (Russell et al., 2012), we focused on the Qinling Mountains at the northern extent of the giant panda distribution. This study aimed to (1) understand the current status and future changes in giant panda habitat range, suitability, and fragmentation in this Qinling Mountains; (2) quantify the scale of habitat shift according to direction and elevation, and describe spatial dynamics of habitat fragmentation resulting from climate change; and (3) provide conservation recommendations for giant panda regarding population management, reserve networks, and habitat restoration.

## 2 Materials and Methods

### 2.1 Study area

**Figure 1:**
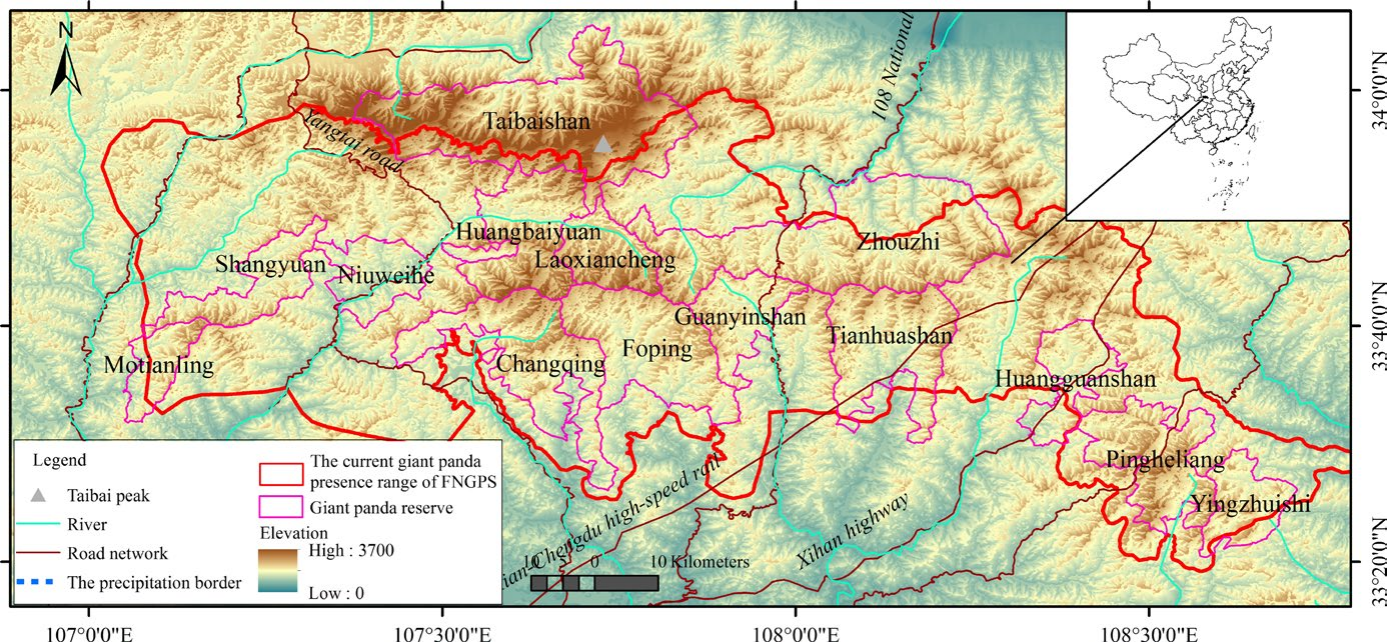
Giant panda population range and nature reserves in the Qinling Mountains, China

The Qinling Mountains are home to 18.6% of wild giant pandas and represent the densest population in China based on the FNGPS. The mountains include 596,681.1 ha of giant panda population range, and the conservation network of 14 reserves is the largest reserve network in China (Figure 1; State Forestry Administration (SFA), 2014). The mountain range forms a watershed between the Yangtze and Yellow rivers and peaks with Taibai Mountain at 3,767 m above sea level. Several major roads run north–south in the study area (national roads 108 and 210, Xihan expressway). We chose the Qinling Mountains for the following reasons. First, these mountains have a transitional climate between northern subtropical and warm temperate zones and represent a typical area to project the impacts of climate change due to high climatic variability. Second, the giant pandas in the Qinling Mountains are geographically and genetically isolated from other populations and this helps simplify the scenarios for population dispersal and climate change. Third, giant pandas inhabiting mountains should be the most sensitive to climate change and trigger a habitat change or migration based on future scenarios and the edge effects of ecology, high latitude, and high population density (Guralnick, 2006). This area has also been identified as a global biodiversity hot spot and a global conservation priority (Mittermeier, Myers, Mittermeier, & Robles Gil, 2002; Olson & Dinerstein, 1998). Due to the great value of this ecosystem, this area is designated as a major ecological function‐oriented zone to protect its ecological function, nature forest protection area to protect its forest resource, and water storage area to protect water resource by the Chinese central government (Fan, Tao, Qing, & Ren, 2010; Yue, Long, Long, & Qian, 2012).

### 2.2 Giant panda presence data and environmental variables

The Chinese State Forestry Administration conducted the FNGPS in the Qinling Mountains from April to June 2012 and recorded 1810 sign points indicating the presence of giant pandas including feces, dens, sleeping sites, and footprints (Table 1). These data were collected at a frequency of one transect per 200 ha across the giant panda range. Latitude, longitude, elevation, slope, vegetation, and bamboo cover at each sign point were recorded. We derived elevation and slope data from a digital elevation model based on 1:50,000 topographic maps obtained from the Chinese Academy of Sciences (www.gscloud.cn). Vegetation with 80% accuracy and bamboo with 70% accuracy in the study area were obtained from the survey and satellite images of Landsat 5 in 2000 and Spot5 in 2012 using the maximum likelihood classification algorithm in supervised classification by Erdas 9.2 (Leica Geosystems GIS and Mapping, 2003, LLC, Atlanta, GA, USA). All above data were applied and approved by the Chinese State Forestry Administration. As the main human‐induced threats to giant pandas (Gong, Meng, Chen, & Song, 2012; Zhu, Hu, Zhang, & Wei, 2013), human communities and the latest road data, including national roads, highways, and high‐speed railways, were taken from previous studies and field surveys.

**Table 1:** The source and accuracy of study data with its modeling assumption

Data	Source	Accuracy	Modeling assumption
Giant panda occurrence	FNGPS of SFA	100%	
Vegetation	FNGPS and image classification	80%	Tree line increase 30 in south and 14 in north of Qinling mountains
Bamboo	FNGPS and image classification	70%	Keep stable
Elevation	Chinese Academy of Science		Keep stable
Slop	Chinese Academy of Science		Keep stable
Resident community	Field survey	100%	Keep stable
Road network	Field survey	100%	Keep stable
Current climate data	IPCC (www.worldclim.com)	30s	
2050 climate data	IPCC (www.worldclim.com)	30s	Change projected by IPCC (Hijmans et al., 2005)

All geospatial data were based on the UTM WGS 84 coordinate system. The raster data resolution was 30 × 30 m, and data were analyzed using ArcGIS10.0 (Esri, Redlands, CA, USA).

### 2.3 Climate data

We obtained current and future bioclimatic variables at a 30‐s resolution from the WorldClim database (WorldClim.com), and repartition it as grid data with 30 × 30 m size based on its climatic value for matching the resolution of other habitat variables in advance. We only modeled habitat suitability for giant pandas under the climate change scenarios of 2050 to make the projections more effective and applicable on current and impending conservation practices at an expected temporal scale. To minimize collinearity for modeling among 19 bioclimatic variables in the general circulation model (GCM), we employed eight climatic variables (Bio 2, Bio 4, Bio 10, Bio 11, Bio 15, Bio 17, Bio 18, and Bio 19; Table 4) to represent climate conditions in 2050 by removing one variable when the correlation coefficient >|0.70| based on intercorrelation testing among two of the 19 bioclimatic variables (Li et al., 2014). Among 19 GCMs in WorldClim, BCC‐CSM1‐1, CCSM4, HadGEM2‐ES, and MIROC5 were adopted as future climate change projection models based on the GCMs used in previous studies of climate impacts on giant pandas (Li et al., 2014; Liu, Guan, Dai, Li, & Gong, 2016). To avoid the uncertainty of future climate projections from different GCMs, we averaged the value of eight climatic variables of 2050 projected under four selected GCMs using ARCGIS 10.0, and used the average value to form the future climatic raster with 30 × 30 m resolution and to construct climate models. Due to a ban on large‐scale industrial projects in the future within our study area by central and local governments (Jie, Wei, Kan, & Dong, 2012), the developing model with low CO_2_ emissions will be the most possible strategy for the future economy in this study area. So we selected the representative concentration pathways (RCP) of 2.6 as the future emissions scenarios (Hijmans, Cameron, Parra, Jones, & Jarvis, 2005; Parry, Canziani, Palutikof, Linden, & Hanson, 2011).

### 2.4 Modeling

We chose the maximum entropy modeling approach implemented in Maxent (Phillips & Dudík, 2008) to identify current habitat suitability, the importance of permutation, the contribution of each environmental variable, and future status under climate change by 2050. This model is considered as a typical method for habitat suitability modeling of the impacts of climate change by associating presence‐only data, habitat variables, and climatic variables (Gallagher, Hughes, & Leishman, 2013). Due to occurrence bias across giant panda sign points, we thinned the set of occurrence trails with the delete identical function in ArcGIS 10.0 based on 1,800 m tolerance (the radius of a giant panda home range in Qinling; Pan, Mcshea, Garshelis, Wang, & Harris, 2014), to reduce the possibility of the model overestimating environmental conditions at sites of sign clustering and underestimating environmental conditions in areas with low sampling density. After bias removal, the calibration points consisted of 465 sign points from 1,810 trial records. To further characterize model performance, we calculated the average test values of the area under the curve (AUC) with different random subsamples (70% training and 30% test data). The AUC value is widely used as an indicator of a model's ability to discriminate between suitable and unsuitable habitats (Dan & Seifert, 2011) and the models were considered reliable when AUC > 0.75 (Rebelo, Tarroso, & Jones, 2010). Permutation importance depends on the final model and is better for evaluating the importance of a particular variable (Songer et al., 2012). Therefore, we evaluated the importance of the habitat variables based on permutation importance.

The main environmental variables incorporated into the simulation model were vegetation, bamboo, elevation, slope, resident community, and road disturbances including current and 2050 climatic variables (Table 1), based on previous studies of habitat selection (Feng, Manen, Zhao, Li, & Wei, 2009; Hu, 1987; Liu et al., 2014; Qi et al., 2012). Due to technical limitations and the complexity of prediction, future vegetation and bamboo for our study were derived at using following assumptions:

1. The location of the tree line ecotone is considered as a particularly sensitive bioclimatic indicator of climate and landscape change (Holtmeier & Broll, 2005), and climate change will drive the tree line upslope and poleward (Hansen, 2001; Kittel, Steffen, & Iii, 2010; Theurillat & Guisan, 2001). Due to the complexity of projecting the change in the tree line under climate change by models and parameter setting, we made a rough estimation based on the results of a previous study of tree lines in the Qinling Mountains (He, 2014). According to the average increase in the tree line by 16.7 m south and 7.6 m north in the Qinling Mountain from 1988 to 2009 by He (2014), the annual rate of tree line growth was estimated as 0.79 m/a in south and 0.36 m/a in north just based on temporal scale. Then, we predicted the tree line would keep growing 30 m south and 14 m north from 2012 to 2050, and made an upward buffer with a distance of 30 m (south) and 14 m (north) in the Qinling Mountains along the boundary of current high elevation forest based on a vegetation map from 2012, and produced a vegetation map for 2050 modeling.
2. Although bamboo is the main food source for giant pandas, bamboo and flowering stochasticity has not devastated giant panda genetic diversity historically because this species has evolved a series of adaptive strategies to switch bamboo species and disperse long distances to forage (Zhu, Hu, Qi et al., 2013), including 314,807 ha of bamboo in the Qinling Mountains from FNGPS. Furthermore, bamboo always occurs under forests or mixes with other types of vegetation, and its classification and mapping face technical challenges and no basis for projecting dispersal range is available (Tuanmu et al., 2013; Wang et al., 2010). With enough spatial range and limitations estimating dynamics, we integrated bamboo into habitat factors but assumed that bamboo remained stable during habitat prediction modeling.
3. According to the zoning as major ecological function‐oriented zone and water resource conservation area, we also made an assumption of modeling on the human disturbance that resident community and road network will keep static by banning large‐scale construction of township or community and implementing migration projects of impoverished people living in remote area by government in 2005 (Table 1; Jie et al., 2012).

To further validate our model, the study area was divided into 2‐km^2^ grids based on the size of transects during the giant panda survey (SFA, 2014), and the relationship between the number of giant panda trails (indicating habitat selection preference) and the mean habitat suitability index values within each grid was analyzed using Pearson's correlation in SPSS 18.0 (SPSS Inc., Chicago, IL, USA).

### 2.5 Habitat suitability, shift, and fragmentation assessment

We ran the Maxent model with current and future climatic and environmental variables to assess current habitat suitability and to project changes by 2050. All current and future habitats were divided into ordinary, subsuitable, and suitable based on the habitat suitability index. Using expert knowledge and experience in the identification of habitat suitability (Wood & Dragicevic, 2007), we calculated the percentage of giant panda signs in each type of habitat to indicate suitability. Then, we created criteria for habitat suitability assessment and set the habitat suitability index as follows: Habitat with 70% of the giant panda signs was suitable, habitat with 20% of the signs was subsuitable, and other habitat was ordinary. We were able to assess habitat suitability and spatial variations under climate change in 2050 using the proportions and spatial pattern of suitable and subsuitable habitats.

In order to quantify the shift in habitat spatial pattern and fragmentation, patches of suitable and subsuitable habitat were used to analyze dynamics. Latitude, longitude, and elevation at the centroid of all suitable and subsuitable habitat patches between now and 2050 were used to quantify how habitat suitability will shift in direction and elevation using independent samples testing in SPSS 18.0. To understand habitat fragmentation across the landscape, we grouped suitable and subsuitable habitats together and calculated the following indices to assess the dimension of fragmentation (O'Neill et al., 1988): total area (TA); patch number (PN); patch density (PD, number of patches/100 ha in TA); mean patch size (MPS); and largest patch size (LPS). Considering the upward impacts on elevation of climate change on wildlife (Hole et al., 2009), and further identification of the range of high‐quality habitat loss and fragmentation change in elevation gradient under climate change, all current and future suitable habitat patches were used as the indicator and grouped at an elevation interval of 100 m. We then calculated total size, mean size, and the largest size of habitat patch type every 100 m of elevation and compared changes associated with elevation gradient. Given our aim to quantify shifts in habitat and fragmentation, we restricted the area for which we compared habitat patches within the giant panda distribution as defined by the FNGPS (State Forestry Administration (SFA), 2014).

## 3 Results

### 3.1 Habitat suitability shifts under future climate scenarios, model validation, and variable importance

**Figure 2:**
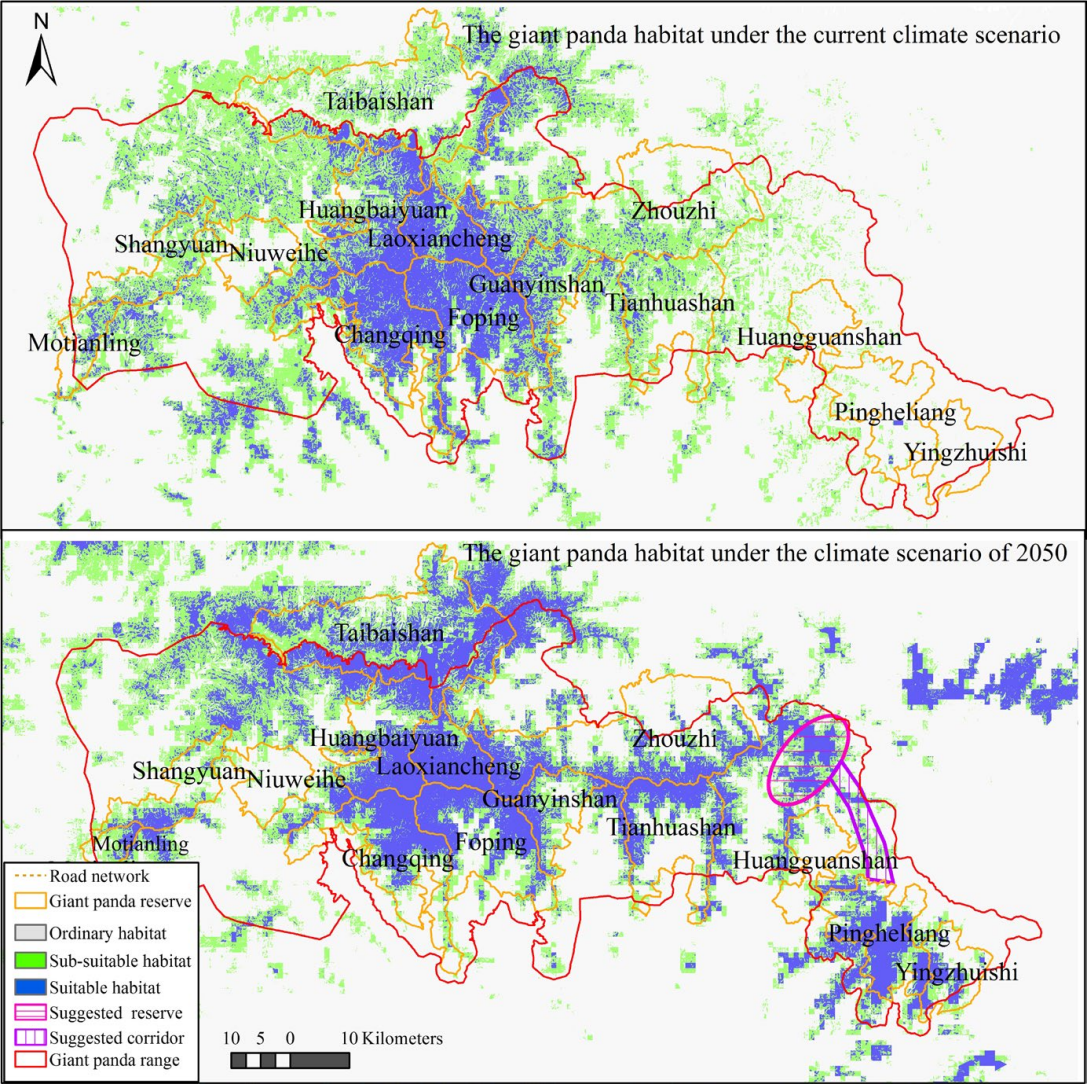
Changes in giant panda habitat under current and 2050 climate scenarios

Based on habitat classification criteria, the index of suitable habitat was 0.5–1, the index of subsuitable habitat was 0.2–0.5, and the index of ordinary habitat was 0–0.2. The area of giant panda habitat suitability is not predicted to undergo significant variation under climate change by 2050 based on the proportion of suitable and subsuitable habitats (Table 2; Figure 2). However, suitable habitat and subsuitable habitat will be affected in opposite ways: 9.1% increase in suitable habitat and 9.0% decline in subsuitable habitat because of climate change. Comparing the latitude, longitude, and elevation of habitat patch centroids, habitat suitability undergoes significant spatial change northward, eastward, and upward. Suitable habitat will expand 8 m upward (*Z* = −7.3, *p* < .05) and 129 m northward (*Z* = −3.8, *p* < .05), and subsuitable habitat will expand 38 m upward (*Z* = −14.2, *p* < .05) and 2,039 m northward (*Z* = −16.3, *p* < .05). We also found a major eastward shift in habitat suitability including 11,110 m (*Z* = −15.8, *p* < .05) for suitable habitat and 2,482.8 m (*Z* = −6.2, *p* < .05) for subsuitable habitat.

**Table 2:** Current and 2050 giant panda habitat suitability, proportion induced by climate change, including mean latitude, longitude, and elevation of centroids for three types of habitat patches

Habitat type	The current					2050 (Rcp2.6)				
	Area (ha)	Proportion (%)	Mean longitude (m)	Mean latitude (m)	Mean elevation (m)	Area (ha)	Proportion (%)	Mean longitude (m)	Mean latitude (m)	Mean elevation (m)
Suitable	121148.7	20.30	745375.0	3736643.6	1904.8	175312.1	29.40	756485.4	3736772.8	1912.9
Subsuitable	197580.8	33.10	758012.2	3733184.0	1936.7	143784	24.10	760495.0	3735223.9	1974.9
Ordinary	277951.6	46.60	747965.5	3735007.5	1902.5	277585	46.50	755533.2	3735530.9	1914.2

Both the training AUC (0.91) and test AUC (0.87) indicated reliable model performance (Table 3). Additionally, the correlation between the presence of giant pandas and habitat suitability index was significant (ρ = 0.56, *p* < .05), confirming that our methods captured the relationship between giant panda occurrence and habitat spatial distribution under current climatic conditions. The most important climate variable based on permutation importance was temperature seasonality (Bio4, 25%), followed by precipitation seasonality (Bio15, 23%). Slope was the most important habitat variable (5.8%) among abiotic and biotic habitat factors after climate factors. The disturbance on giant panda habitat from transportation is much higher than disturbance arising from resident community (Figure 2).

**Table 3:** Bioclimatic variables and their contribution and percent permutation importance reported by Maxent. Variables are in the order of highest to lowest permutation importance

Habitat variables	Description	Variable contribution (%)	Permutation importance (%)
Bio4	Temperature seasonality	22.9	25.0
Bio15	Precipitation seasonality	20.4	23.0
Bio11	Mean temperature of coldest quarter	5.4	16.3
Bio17	Precipitation of driest quarter	7.4	8.4
Slop	Topographic characteristic	6.8	5.8
Bio18	Precipitation of warmest quarter	14.1	4.9
Bio2	Mean diurnal range	9.0	4.2
Elevation	Topographic characteristic	2.1	3.7
Vegetation	Vegetation formation group	1.5	2.4
Bamboo	Food resource	1.2	1.9
Bio19	Precipitation of coldest quarter	0.1	1.3
Road	Anthropogenic disturbance of transportation	3.3	1.1
Aspect	Topographic characteristic	0.8	0.8
Bio10	Mean temperature of warmest quarter	4.5	0.7
Resident	Anthropogenic disturbance of human activity	0.5	0.5

### 3.2 Habitat fragmentation and elevation shift due to climate change

Our landscape indices indicated that fragmentation of habitat will decrease because of climate change by 2050. In particular, as patches of all suitable habitat become more integrated due to climate change, several large patches along the Qinling Mountains ridge from central to eastern regions will form (growth in MPS and LPS), and the landscape pattern of habitat will be more aggregated(Table 4; Figure 2).

**Table 4:** Habitat fragmentation indices of giant panda habitat (suitable and subsuitable) under current and 2050 climate scenarios

Indices	Current habitat	2050 habitat
TA (ha)	318729.5	319096.1
PN	28643	26417
PD	9.0	8.3
MPS (ha)	11.1	12.1
LPS (ha)	70799.0	86014.1

TA, total area of habitat; PN, habitat patch number; PD, habitat patch density, number of patches/100 ha in TA; MPS, mean size of habitat patches; LPS, size of the largest habitat patch.

Climate will greatly affect the suitable habitat elevation pattern. Current suitable habitat is mainly around 2,100 m above sea level, around which 61% of total current suitable habitat occurs; future suitable habitat is mainly around 2,200 m above sea level and represents 60% of total future suitable habitat. The majority of suitable habitat loss will occur under 1,900 m from 32,678.1 ha (current) to 16,102.0 ha (by 2050) with 51% decline, and suitable habitat above 1,900 m will noticeably increase from 88,470.6 ha (current) to 159,210.1 ha (by 2050) with 80% growth following climate change (Table 5; Figure 2). MPS and LPS dynamics for suitable habitat elevation showed similar qualitative and quantitative changes regarding fragmentation and loss as for TA, suitable habitat patch size will increase and become more concentrated with a distinct drop in landscape fragmentation at 2,000–2,400 m above sea level (also the most preferred altitude of giant pandas).

**Table 5:** The elevation pattern of current and future (2050) suitable habitats

Elevation range	Current suitable habitat			2050 suitable habitat		
	TA (ha)	MPS (ha)	LPS (ha)	TA (ha)	MPS (ha)	LPS (ha)
1,100	0.1	0.1	0.1	0	0	0
1,200	46.5	1.9	14.7	0	0	0
1,300	174.7	1.3	14.1	1.4	0.5	0.6
1,400	887.8	2.5	78.3	54	1.1	9.9
1,500	2801	3.4	311.4	899.1	6	176.1
1,600	4442.9	3.6	244	1704.6	3	145
1,700	8157.4	4.9	581.2	2320.8	2.5	99.7
1,800	7420.4	4.4	720.2	4963.1	2.9	414.9
1,900	8747.4	5.5	3241.2	6159.1	3.9	1112.6
2,000	4097.3	3.4	134.7	29855.1	23.9	21540
2,100	73661	86.5	70800	6811.8	8.4	1907.6
2,200	4309	7.5	645.7	105504.9	270.5	86010
2,300	1634.1	4.2	160.3	1194.4	7.5	339.6
2,400	2223.2	9.4	1203.1	15754.4	225.1	13290
2,500	1575.6	8.9	261.8	34.1	2.1	17.8
2,600	496.3	3.9	97.5	4.4	0.4	1.4
2,700	232.9	2.7	97.8	2.1	0.2	0.8
2,800	153.1	2.8	26.1	3.4	0.2	0.7
2,900	79.6	1.2	11.4	9.3	1	4.2
3,000	5.5	0.6	2	16.5	1	3.2
3,100	3	1.5	1.8	19.7	3.9	13.2

TA, total area of habitat; MPS, mean size of habitat patches; LPS, size of the largest habitat patch.

## 4 Discussion

In general, wildlife population ranges shift toward the poles and to higher elevations under climate change conditions (Hole et al., 2009). Our results are coincided with previous studies at the spatial shift of giant panda habitat in northward, upward, and northwestward (Fan et al., 2014; Liu et al., 2016; Tuanmu et al., 2013), but vary a great deal as following: (1) The significant loss of habitat was not detected compared to the results of Songer et al. (2012) and Fan et al. (2014); (2) the further fragmentation of habitat was not projected supporting the finding of Songer et al. (2012) and opposing the results of Shen et al. (2015); (3) we firstly quantify the spatial shift of habitat by climate change and found an additional predicted an 11‐km eastward shift for giant panda habitat by 2050 which was not reported by previous studies. The reason for such contrasting results may be explained by study scale and the quality of variables. All previous studies set their study areas far more big than current giant panda population range a, but we just restricted our study area within current population range from the FNGPS, and the data of giant panda occurrence for habitat modeling are still within the region currently used by giant panda. So there maybe is a spatial autocorrelation from the bias of distribution in calibration data to cause the great loss out of current population range during habitat projection. Due to the change in giant panda population pattern in Qinling since 2000 (Gong et al., 2016), the latest occurrence data from the FNGPS in our study can better associate the relationship between presence and habitat variables including climate factors. Further, to lessen the uncertainty of projection results by different GCMs, the value of climate variables for modeling in our study was averaged based on used GCMs. The high AUC value in the Maxent model proved our results were reliable, the variable contribution and permutation importance of habitat factors in our study were consistent with previous studies of climate impacts on giant pandas (Liu et al., 2016; Songer et al., 2012), and the results from giant panda habitat selection studies that found fidelity for specific slopes override elevation and aspect because of the need to maintain balance between energy intake and expenditure (Hu, 1987; Schaller, 1985; Zhang et al., 2011). Moreover, our study provides a methodological case to analyze future climate variables with better accuracy and can be applied to other climate change studies.

Habitat fragmentation has always been the main threat to giant pandas (Gong, Yang, Yang, & Song, 2010) and climate change may pose an additional challenge for conservation. In addition to further fragmentation of subsuitable habitat, climate change will increase risks to giant panda safety. For example, while suitable habitat (>1,900 m elevation) will expand and become more connected because of climate change, areas of lower elevation (<1,900 m elevation) will not be suitable for giant pandas due to exacerbated habitat fragmentation and loss (Figure 2). This spatial shift in suitable habitat may force giant pandas to move to higher elevations and result in overcrowding. Based on the current population density of giant pandas from the TNGPS and FNGPS, the population across seven reserves (Taibaishan, Huangboyuan, Laoxiancheng, Zhouzhi, Changqing, Foping, and Guanyinshan) in the central Qinling Mountains has increased from 203 to 237 since 2000 (SFA 2006, 2014), from which it can be inferred that a habitat shortage will most likely occur in these areas. Due to the same consideration on carrying capacity locally, giant pandas have often been seen entering the area used by human, such as villages and agricultural land in the central Qinling Mountains (Sina, 2013; Xinhuanet, 2015). Population safety and risk monitoring programs should be implemented or expanded in this area to respond to habitat fragmentation and loss induced by climate change in the future.

Coinciding with the results of Zhang et al. (2014) suggesting spatial change eastward, recent monitoring data of WWF (2015), and the dynamics of population pattern (Gong et al., 2010), we found an eastern habitat shift and determined the elevation range patterns of habitat fragmentation under climate change. Despite the challenge of habitat loss, fragmentation, and shift due to climate change, the 54,163 ha of new climate‐suitable habitat in the eastern Qinling Mountains will accommodate no less than 51 giant pandas based on basic home range requirements (10.62 km^2^)and provides hope for population expansion (Pan et al., 2014). Actually, giant pandas in the eastern reserves currently face a high probability of local extinction due to small population size and poor genetic diversity, and it is necessary to increase the population size via translocation or the reintroduction of captive individuals.

The changes in habitats modeled here mean that a new giant panda conservation plan is needed to respond to climate change. Due to habitat integrity at the landscape scale (Loucks et al., 2001), the northeast part of the Qinling Mountains may become suitable habitat, and as it is currently outside the current reserve network, it should be included in current conservation planning and zoning (Figure 2). A new habitat restoration program is also needed to welcome the climate change‐driven migration of giant pandas by 2050 (Figure 2). Last, a conservation corridor between the large patch of future suitable habitat in the northeast and southeast Qinling Mountains should be built to link future habitats and facilitate population dispersal.

## Conflict of Interest

None declared.
